# Octogenarian patients with colon cancer – postoperative morbidity and mortality are the major challenges

**DOI:** 10.1186/s12885-022-09384-9

**Published:** 2022-03-21

**Authors:** Øystein Høydahl, Tom-Harald Edna, Athanasios Xanthoulis, Stian Lydersen, Birger Henning Endreseth

**Affiliations:** 1 Department of Surgery, Levanger Hospital, Nord-Trøndelag Hospital Trust, Levanger, Norway; 2 IKOM Department of Clinical and Molecular Medicine, NTNU, Norwegian University of Science and Technology, Trondheim, Norway; 3 Regional Centre for Child and Youth Mental Health and Child Welfare – Central Norway, Department of Mental Health, Faculty of Medicine, Norwegian University of Science and Technology, Trondheim, Norway; 4 Clinic of Surgery, St. Olavs Hospital, Trondheim University Hospital, Trondheim, Norway

**Keywords:** Colon cancer, Octogenarians, Treatment, Survival, Epidemiology

## Abstract

**Background:**

Few studies have addressed colon cancer surgery outcomes in an unselected cohort of octogenarian patients. The present study aimed to evaluate the relative survival of octogenarian patients after a major resection of colon cancer with a curative intent.

**Methods:**

All patients diagnosed with colon cancer at Levanger Hospital between 1980 and 2016 were included. We performed logistic regression to test for associations between 90-day mortality and explanatory variables. We performed a relative survival analysis to identify factors associated with short- and long-term survival.

**Results:**

Among 237 octogenarian patients treated with major resections with curative intent, the 90-day mortality was 9.3%. Among 215 patients that survived the first 90 days, the 5 year relative survival rate was 98.7%. The 90-day mortality of octogenarian patients was significantly higher than that of younger patients, but the long-term survival converged with that of younger patients. Among octogenarian patients, the incidence of colon cancer more than doubled during our 37-year observation period. The relative increase in patients undergoing surgery exceeded the increase in incidence; hence, more patients were selected for surgery over time. A high 90-day mortality was associated with older age, a high American Society of Anaesthesiologists (ASA) score, and emergency surgery. Moreover, worse long-term survival was associated with a high Charlson Comorbidity Index, a high ASA score, a worse TNM stage, emergency surgery and residual tumours. Both the 90-day and long-term survival rates improved over time.

**Conclusion:**

Among octogenarian patients with colon cancer that underwent major resections with curative intent, the 90-day mortality was high, but after surviving 90 days, the relative long-term survival rate was comparable to that of younger patients*.* Further improvements in survival will primarily require measures to reduce the 90-day mortality risk.

## Background

Colon cancer mainly occurs among older individuals. In Nordic countries, increases have been observed in the population, life expectancy, and incidence of colon cancer over the last few decades. These trends are likely to continue; thus, the number of older patients with colon cancer will continue to increase [1, 2], and a significant proportion of these patients will be octogenarians (i.e., aged 80–89 years) [3, 4].

In Norway, a standardized evidence-based approach to assessing and treating colon cancer has been established at a national level [5]. The final treatment strategy for an individual patient should be based on an accurate staging of the disease and on patient-related factors. The national guidelines recommend that multidisciplinary teams undertake treatment decision-making. Guidelines related to adjuvant chemotherapy administration and follow-up times are recommended according to the patient’s chronological age.

In the literature, the group of older patients with colon cancer is a vaguely defined term. The definition of ‘older age’ ranges from ≥ 65 to > 80 years [6–8]. The mainstay of treatment for colon cancer is radical surgery, and this is combined with chemotherapy in selected subgroups of patients. Previous reports have noted that both radical surgery and chemotherapy are increasingly underused with increasing patient age [7, 9]. Despite some variation, several studies have also reported that postoperative morbidity and mortality increased with increasing age [10, 11]. Although it is well known that, overall, long-term survival decreases with increasing age, estimates of long-term disease-free and relative survival rates have varied for older patients treated for colon cancer.

The present study aimed to evaluate the trends, treatments, and outcomes observed over a period of nearly four decades in patients diagnosed with colon cancer. In particular, we investigated octogenarian patients. Over time, this heterogeneous group of patients has become larger. Therefore, it is of paramount importance to raise our awareness of patient-related factors and their impact on cancer treatment outcomes in older patients. Based on this knowledge, we can establish evidence-based, individualized treatment strategies.

## Methods

This study included 1530 consecutive patients admitted with colon cancer at Levanger Hospital during 1980–2016. Levanger Hospital is the primary hospital of 10 municipalities in Norway, and the catchment area remained unchanged throughout the study period. The population increased by 18%, from 83,890 inhabitants in 1980, to 99,566 inhabitants in 2016. During this period, the average age of the population also increased. In particular, the number of octogenarian inhabitants increased by 73%, from 2184 individuals in 1980, to 3800 individuals in 2016 [4].

Through the hospital administrative system, we accessed the health records for all patients that were discharged with diagnosis codes of the International Classification of Diseases, 8^th^ revision (ICD-8) from 153.1 to 153.9, with ICD-9 codes from 153.0 to 153.9, and with ICD-10 codes from C18.0 to C19. Data on all patients were recorded, crosschecked, and confirmed with data from the Norwegian Cancer Registry, during 1980–2016. From the hospital database, we retrieved data on demographic and logistic variables, comorbidities, treatment, tumour characteristics (including histopathology), complications after treatment, and short- and long-term outcome measures.

We defined colon cancer as any tumour located above 15 cm from the anal verge. Right colon tumours were defined as tumours localized in the caecum, ascending colon, hepatic flexure, or transverse colon. Left colon tumours were defined as tumours localized in the splenic flexure, descending colon, or sigmoid colon. Tumours located within 15 cm from the anal verge were defined as rectal cancer, and we excluded these and cancers localized in the appendix.

We characterized patient comorbidity with the American Society of Anaesthesiology (ASA) score and the Charlson Comorbidity Index (CCI) [12, 13]. We defined anaemia at admission, as advocated by the World Health Organization, as blood haemoglobin levels below 13 g/dL in males and below 12 g/dL in females [14]. We also defined “moderate to severe” anaemia as haemoglobin levels below 11 g/dL in males and 10 g/dL in females. Surgical complications were defined according to the Clavien-Dindo classification of surgical complications, grades I-V [15]. Surgical complications were recorded as in-hospital complications from the day of admission to the day of discharge.

Disease stages were based on the TNM classification, sixth edition [16]. An R0 resection was defined as no detectable residual tumour postoperatively; an R1 resection was defined as a microscopic residual tumour detected in a postoperative histological examination; and an R2 resection was defined as a macroscopic residual tumour detected after surgical treatment [17]. An R0 resection was further classified into two groups: an R0 without tumour perforation and an R0 with tumour perforation. Tumour perforations included both spontaneous (12) and iatrogenic perforations (9).

Patients were categorized into five groups, according to treatment intent: (*i*) a major resection with curative intent (R0 and R1), (*ii*) a polypectomy, (*iii*) a major resection with non-curative intent, (*iv*) a bypass/stoma, and (*v*) best supportive care.

Emergency surgery was defined as surgery due to evidence of a large bowel obstruction or large bowel perforation. The laparoscopic colon resection technique was gradually introduced during the last part of the study period. A total of 49 patients underwent laparoscopic surgery. In ten of these patients, the procedure was converted to open surgery.

Staging varied throughout the observation period. Staging was based on complete clinical and histopathological examinations of the resected specimen in 84.9% (1299/1530) of patients; a clinical examination and histopathological examination of a tumour biopsy in 7.8% (120/1530) of patients; a pathological evaluation during an autopsy in 1.4% (21/1530) of patients, and clinical evaluations alone in 5.9% (90/1530) of patients.

Since 1993, the Norwegian national guidelines for treatment of colon cancer advocated that all patients aged 75 years or under with Stage III disease should be evaluated for adjuvant chemotherapy. Later, this recommendation was applied to selected patients with Stage II disease [5].

Follow-ups were initially conducted according to local guidelines. Starting in 1993, they were based on very similar, national guidelines [5]. The follow-up time was calculated as the patient-years at risk, starting from the date of admission. The study endpoints were: local recurrence, metastasis, or death, regardless of cause. The mean follow-up time was 6.05 years (standard deviation [SD] = 6.89, range: 0–38.7 years). The end of follow-up was December 31st, 2018.

### Statistical analyses

The Exact Unconditional z-pooled test was used to compare binomial proportions; for example, the percentage of reoperations, relative to the percentage of emergency or elective primary operations. The Cochran Armitage exact trend test was used to test for trends in proportions; for example, the proportions of elective surgeries vs. emergency surgeries in different age groups. The Joncheere-Terpstra test was used to test for the distribution of age, as a dependent variable, across 10-year age groups, as the independent variable. The 5 year rates of local recurrence and metastases were estimated with the Kaplan–Meier method.

Logistic regression analyses were performed to assess associations between the 90-day mortality, as the dependent variable, and different explanatory variables. Ordinal logistic regressions were performed to analyse the associations in doubly-ordered r × c tables; for example, the ASA score stratified by age group. The resulting odds ratios (ORs) represent a common OR estimate for any 2 × 2 table that would occur, if the r × c table was collapsed to a 2 × 2 table, based on any cut-off threshold, along the columns and rows. Multinomial logistic regression analyses were performed in singly ordered r × c tables; for example, the type of treatment, stratified by age groups.

#### Relative survival analysis

Relative survival was defined as a measure of mortality compared to the general population. The observed survival in the group with cancer was divided by the expected survival of a comparable group in the general Norwegian population, matched by age, sex, and the calendar year of investigation. Relative survival was estimated with the Ederer II method and analysed with STATA 16 [18]. Multivariable analyses were performed with a full likelihood approach. Norwegian population survival probabilities were downloaded from the Human Mortality Database, for every year from 1980, calculated for groups stratified by sex and age [19].

Two-sided p-values < 0.05 were considered significant. Means are reported with the range (minimum to maximum) and SD, where relevant. Ninety-five percent confidence intervals (95% CI) are reported, when relevant. Analyses were carried out in Stata 16, IBM SPSS Statistics 25, and StatXact 9.

## Results

### Study population

Table 1 presents the characteristics of all 1530 patients admitted with colon cancer between 1980 and 2016. There were 750 males (49%) and 780 females, with mean ages of 72.3 (range: 32.9–96.1, SD: 11.1) years and 73.2 (range: 20.3–99.6, SD: 11.6) years, respectively. The mean age of the population increased from 71.5 years, in 1980–1989, to 74.5 years, in 2010–2016 (*p* = 0.001). The mean number of patients admitted per year increased by 109%, from 27.4 patients/y in 1980–1989 to 57.4 patients/y in 2010–2016. The number of octogenarian patients increased by 131%, from 6.7 to 15.5 patients admitted per year, respectively.

**Table 1:** Characteristics of all patients admitted with colon cancer during the 1980–2016 study period

Characteristic	Total, ***n*** = 1530	Age group (years)						*P* value
		< 65, *n* = 353	65–74, *n* = 451	75–79, *n* = 281	80–84, *n* = 269	85–89, *n* = 124	≥ 90, *n* = 52	
Sex								0.031^a^
Females	780 (51)	181 (23)	210 (27)	139 (18)	148 (19)	70 (9)	32 (4)	
Males	750 (49)	172 (23)	241 (32)	142 (19)	121 (16)	54 (7)	20 (3)	
Calendar year								< 0.001^b^
1980–1989	274 (18)	70 (26)	89 (33)	45 (16)	49 (18)	18 (7)	3 (1)	
1990–1999	367 (24)	85 (23)	115 (31)	73 (20)	56 (15)	29 (8)	9 (3)	
2000–2009	487 (32)	129 (27)	121 (25)	85 (18)	100 (21)	33 (7)	19 (4)	
2010–2016	402 (26)	69 (17)	126 (31)	78 (19)	65 (16)	44 (11)	21 (5)	
Charlson Comorbidity Index								< 0.001^b^
0	1076 (70)	300 (85)	321 (71)	187 (66)	165 (61)	75 (61)	28 (54)	
1–2	358 (23)	51 (14)	100 (22)	72 (26)	81 (30)	39 (32)	15 (29)	
> 2	96 (6)	2 (1)	30 (7)	22 (8)	23 (9)	10 (8)	9 (17)	
ASA score								< 0.001^b^
1–2	832 (54)	282 (80)	284 (63)	134 (48)	94 (35)	28 (23)	10 (19)	
3	598 (39)	66 (19)	147 (33)	131 (47)	150 (56)	76 (61)	28 (54)	
4–5	100 (7	5 (1)	20 (4)	16 (6)	25 (9)	20 (16)	14 (27)	
Localization								< 0.001^a^
Right colon	845 (55)	166 (47)	232 (51)	174 (62)	158 (59)	79 (64)	36 (69)	
Left colon	685 (45)	187 (53)	219 (49)	107 (38)	111 (41)	45 (36)	16 (31)	
Stage (TNM)								0.14^d^
I	189 (12)	42 (12)	61 (14)	34 (12)	34 (13)	15 (12)	3 (6)	
II	582 (38)	127 (36)	160 (36)	117 (42)	101 (38)	47 (38)	30 (58)	
III	331 (22)	78 (22)	120 (27)	62 (22)	54 (20)	12 (11)	5 (10)	
IV	377 (25)	102 (29)	102 (23)	60 (21)	66 (25)	35 (27)	12 (23)	
Unknown	51 (3)	4 (1)	8 (2)	8 (3)	14 (5)	15 (12)	2 (4)	
Treatment intent categories								< 0.001^c^
* Curative intent*								
Major resection^f^	1034 (68)	239 (68)	328 (73)	204 (73)	172 (64)	67 (54)	24 (46)	
Polypectomy	38 (3)	10 (3)	11 (2)	11 (4)	6 (2)	0	0	
* Non-curative intent*								
Major resection	220 (19)	62 (18)	64 (14)	39 (14)	38 (14)	14 (11)	3 (6)	
Bypass/stoma	67 (4)	17 (5)	17 (4)	6 (2)	14 (5)	11 (9)	2 (4)	
* Best supportive care*	171 (11)	25 (7)	31 (7)	21 (8)	39 (15)	32 (26)	23 (44)	
Surgery								0.005^b^
Elective surgery^e^	1081 (82)	263 (83)	339 (83)	217 (88)	176 (79)	72 (78)	14 (48)	
Emergency surgery	240 (18)	55 (17)	70 (17)	32 (12)	48 (21)	20 (22)	15 (52)	

Values are the number of patients (%), unless otherwise indicated. ^a^Cochran-Armitage exact trend test; ^b^Ordinal logistic regression with age group as covariate; ^c^Multinomial logistic regression with age group as covariate

^d^Ordinal logistic regression with age group as covariate, for known stages; ^e^Including polypectomy; ^f^Including *R0* resection, *R0* resection with perforation and *R1* resection

The mean CCI, the mean ASA score, and the proportion of patients with right-sided colon cancer increased with increasing age. We observed no differences in stages among the age groups. Over time, the percentage of patients diagnosed with stage I or II disease increased from 41%, in 1980–1989, to 58% in 2010–2016 (*p* < 0.001). The number of patients with an unknown stage declined over time and was zero in the last time period (2010–2016).

Overall, 89% (1359/1530) of all patients diagnosed with colon cancer underwent a surgical treatment, including a major resection, a polypectomy, or a palliative procedure. The rate of surgeries decreased as patient age increased. During the study period, the percentage of octogenarian patients that underwent a major resection with curative intent increased over time. It was 54% (36/67), in the first time-period (1980–1989), and 61% (66/108), in the last time-period (2010–2016).

The rate of emergency surgery remained stable over time. However, emergency surgery was required more frequently as patient age increased. The rates were 16% (157/976) among patients younger than 80 years and 22% (68/316) among octogenarian patients. The mean hospital stay after a major resection with curative intent decreased from 17.0 days (range: 2–67, SD: 11.7) during 1980–1989 to 9.7 days (range: 4–47, SD: 6.3) during 2010–2016.

### Mortality within 90 days for all patients

Overall, the 90-day mortality rate after admission was 13.5% (206/1530). The mortality rate increased successively as patient age increased. Mortality rates were 6.5% in patients < 65 years, 22.4% in octogenarian patients, and 44.2% in patients above 90 years (*p* < 0.001). During 1980–1989, 21.2% (58/274) of all admitted patients died within 90 days. In comparison, during 2010–2016, only 10.9% (44/402) of patients died. Table 2 presents the prognostic factors we identified that were associated with mortality within 90 days after admission. The odds of death increased with increasing patient age.

**Table 2:** Factors associated with 90-day mortality for all patients admitted with colon cancer in 1980–2016; *n* = 1530

Factors	Unadjusted odds ratio (95% CI)	*P* value	Adjusted odds ratio (95% CI)	*P* value
Age (years)				
< 65	1 (reference)		1 (reference)	
65—74	1.44 (0.84 to 2.44)	0.18	1.41 (0.74 to 2.69)	0.30
75 – 79	1.78 (1.01 to 3.13)	0.045	1.92 (0.95 to 3.88)	0.070
80—84	3.69 (2.20 to 6.18)	< 0.001	2.64 (1.35 to 6.16)	0.005
85 – 89	5.20 (2.91 to 9.30)	< 0.001	2.42 (1.11 to 5.29)	0.027
≥ 90	11.38 (5.70 to 22.72)	< 0.001	5.85 (2.30 to 14.87)	< 0.001
Calendar year	0.97 (0.96 to 0.98)	< 0.001	0.94 (0.92 to 0.96)	< 0.001
Female sex	0.76 (0.57 to 1.03)	0.072	0.76 (0.52 to 1.13)	0.18
CCI	1.65 (1.39 to 1.95)	< 0.001	1.39 (1.10 to 1.77)	0.006
ASA score				
1–2	1 (reference)		1 (reference)	
3	2.82 (1.98 to 4.05)	< 0.001	1.92 (1.21 to 3.06)	0.006
4–5	21.59 (13.26 to 35.14)	< 0.001	6.94 (3.69 to 13.05)	< 0.001
Anaemia^a^	1.44 (1.06 to 1.96)	0.019	1.19 (0.78 to 1.83)	0.42
Emergency surgery	3.12 (2.23 to 4.35)	< 0.001	4.90 (2.99 to 8.05)	< 0.001
Localization (left vs. right)	0.87 (0.64 to 1.17)	0.35	0.78 (0.52 to 1.18)	0.24
TNM-stage				
I	1 (reference)		1 (reference)	
II	2.72 (1.06 to 6.99)	0.038	1.60 (0.54 to 4.74)	0.39
III	1.17 (0.63 to 4.89)	0.29	1.30 (0.40 to 4.23)	0.67
IV	17.61 (7.06 to 43.92)	< 0.001	5.29 (1.57 to 17.80)	0.007
Unknown	32.71 (11.51 to 92.99)	< 0.001	1.78 (0.46 to 6.86)	0.40
Treatment intent categories				
* Curative intent*				
Major resection	1 (reference)		1 (reference)	
Polypectomy	0.59 (0.08 to 4.43)	0.59	1.59 (0.18 to 13.92)	0.68
* Non-curative intent*				
Major resection	5.03 (3.20 to 7.91)	< 0.001	1.28 (0.55 to 9.98)	0.56
Bypass/stoma	28.80 (16.22 to 50.83)	< 0.001	10.23 (4.03 to 26.00)	< 0.001
* Best supportive care*	19.78 (12.95 to 30.21)	< 0.001	9.56 (4.21 to 21.71)	< 0.001

Results are from a logistic regression analysis, with death within 90 days as dependent variable; unadjusted: analysis performed with one covariate at a time; adjusted: analysis performed with all listed covariates included simultaneously. *CCI* Charlson Comorbidity Index, classified in three levels: 0, 1 and 2 + ; ^a^Anaemia was defined as < 13 g/dL in males and < 12 g/dL in women (based on WHO recommendations)

### Long-term relative survival rates for all patients

**Fig. 1:**
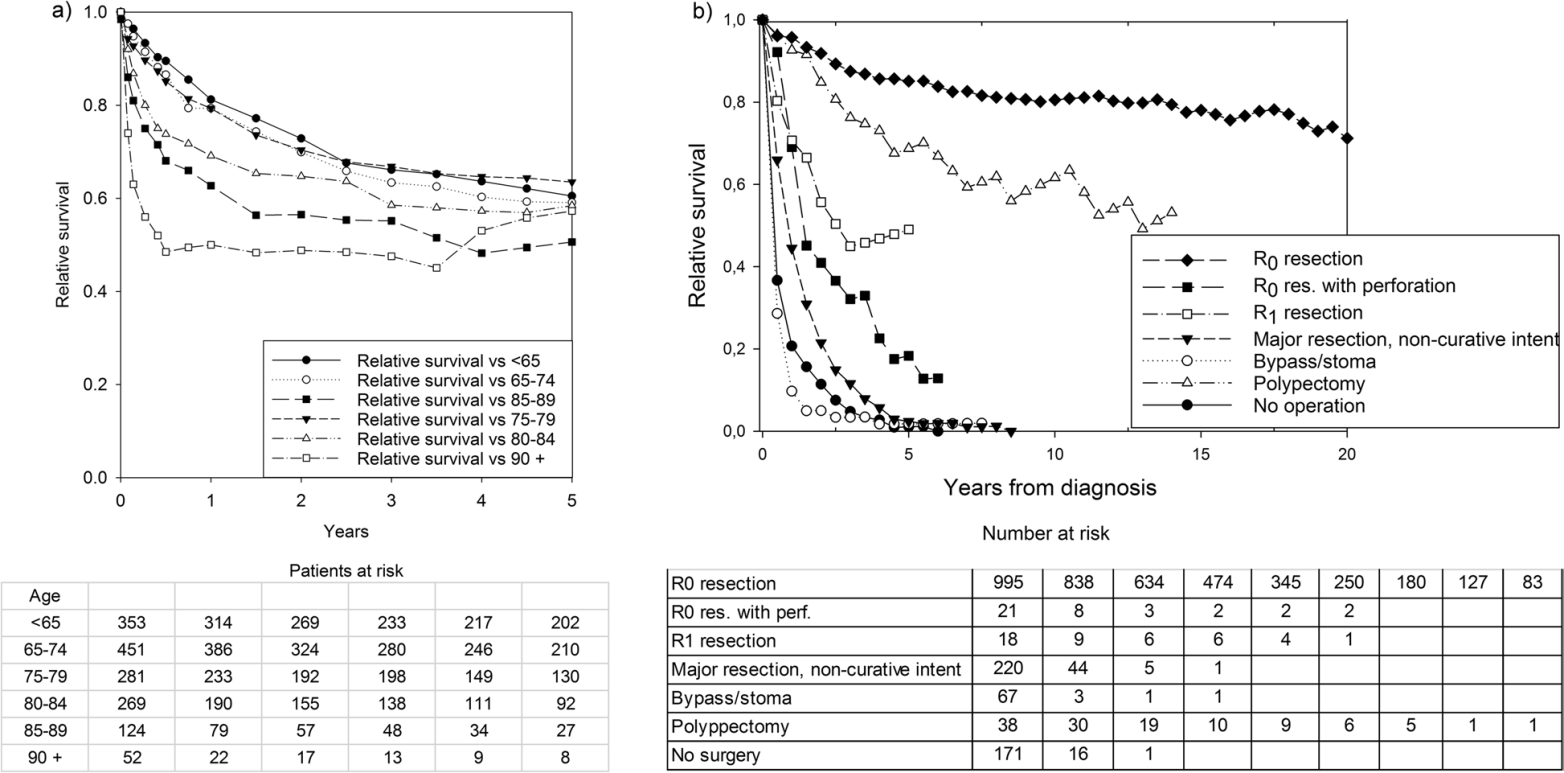
Survival of patients with colon cancer during 1980–2016. **a** 5-year relative survival for all patients in each age group; table columns represent the number of patients at risk at surgery (time = 0) and every 1 year thereafter. **b** Long term relative survival for all patients, classified by treatment intent; table columns represent the number of patients at risk at surgery (time = 0) and every 2.5 years thereafter

Overall, the 5 year relative survival rate for all patients was 58.5% (95% CI: 55.2 to 61.6). Figure 1a presents the 5 year relative survival rates, stratified by age groups. Patients aged 75–79 years had the highest 5 year relative survival rate, at 63.1% (95% CI: 55.2 to 70.6), compared to 55.4% (95% CI: 47.4 to 63.5) in octogenarian patients. Figure 1b presents the relative survival rates stratified by treatment intent categories. The 5 year relative survival rate for the R0 resection group was 85.1% (95% CI: 81.2 to 88.7), compared to 49.1% (95% CI: 22.1 to 75.6) for the R1 resection group, and 18.3% (95% CI: 4.6 to 41.2) for the R0 resection with perforation group. Among patients < 65 years, the 2 year relative survival rates were: 32.6% (95% CI: 21.3 to 44.4) after a major resection with non-curative intent, 0% after a bypass/stoma, and 12.2% (95% CI: 0.3 to 28.1) after the best supportive care. The corresponding rates in octogenarian patients were: 18.8% (95% CI: 8.8 to 32.3) after a major resection with non-curative intent, 0% after a bypass/stoma, and 18.1% (95% CI: 9.3 to 29.8) after the best supportive care.

After excluding patients that died within the first 90 days, the overall 5 year relative survival rate was 67.2% (95% CI: 63.7 to 70.6). Patients aged < 65 years had the lowest 5 year relative survival rate, at 64.4% (95% CI: 58.7 to 69.6), compared to 71.0% (95% CI: 61.3 to 80.6) in octogenarian patients. Table 3 presents the prognostic factors we identified that were associated with long-term relative survival, among patients that survived 90 days after admission.

**Table 3:** Factors associated with long-term relative survival in 1324 patients that survived 90 days after admission

Factors	Unadjusted hazard ratio	*P* value	Adjusted hazard ratio	*P* value
	(95% CI)		(95% CI)	
Age (years)				
< 65	1 (reference)		1 (reference)	
65—74	1.05 (0.81 to 1.36)	0.69	1.22 (0.94 to 1.61)	0.12
75—79	0.93 (0.67 to 1.29)	0.66	1.16 (0.83 to 1.62)	0.37
80 – 84	1.02 (0.71 to 1.46)	0.91	0.92 (0.64 to1.33)	0.65
85 – 89	1.36 (0.83 to 2.25)	0.23	0.86 (0.52 to 1.42)	0.56
≥ 90	1.26 (0.44 to 3.60)	0.67	1.17 (0.56 to 2.47)	0.68
Female sex	1.14 (0.92 to 1.41)	0.23	1.28 (1.04 to 1.58)	0.021
Calendar year				
1980–1989	1 (reference)		1 (reference)	
1990–1999	0.95 (0.70 to 1.29)	0.74	0.68 (0.50 to 0.93)	0.016
2000–2009	0.69 (0.51 to 0.93)	0.016	0.55 (0.40 to 0.74)	< 0.001
2010–2016	0.58 (0.42 to 0.82)	0.002	0.43 (0.31 to 0.61)	< 0.001
CCI	1.36 (1.20 to 1.55)	< 0.001	1.24 (1.07 to 1.43)	0.004
ASA score				
1–2	1 (reference)		1 (reference)	
3	1.32 (1.06 to 1.65)	0.013	1.27 (0.98 to 1.64)	0.066
4–5	4.65 (3.07 to 7.04)	< 0.001	1.95 (1.23 to 3.12)	0.005
Emergency surgery	2.09 (1.62 to 2.68)	< 0.001	1.42 (1.09 to 1.86)	0.010
TNM-stage				
I	1 (reference)		1 (reference)	
II	1.75 (0.66 to 4.65)	0.26	1.36 (0.69 to 2.68)	0.37
III	6.61 (2.61 to 16.74)	< 0.001	4.71 (2.47 to 8.99)	< 0.001
IV	44.81 (17.96 to 111)	< 0.001	3.39 (1.68 to 6.85)	0.001
Unknown	24.29 (8.67 to 68.05)	< 0.001	1.48 (0.65 to 3.34)	0.35
Treatment intent categories				
* Curative intent*				
Major resection	1 (reference)		1 (reference)	
Polypectomy	1.62 (0.71 to 3.74)	0.25	3.82 (1.54 to 9.48)	0.004
* Non-curative intent*				
Major resection	16.13 (12.51 to 20.80)	< 0.001	9.50 (5.94 to 15.18)	< 0.001
Bypass/stoma	25.50 (16.59 to 39.20)	< 0.001	19.38 (10.82 to 34.69)	< 0.001
* Best supportive care*	21.22 (15.74 to 28.61)	< 0.001	22.24 (12.26 to 37.28)	< 0.001

Results are from a multivariable analysis; unadjusted: performed with one covariate at a time; adjusted: performed with all listed covariates included simultaneously. *CCI* Charlson Comorbidity Index, classified as 0, 1, or 2 +

## Patients with stage I-III disease that underwent a major resection with curative intent

Table 4 presents the characteristics of all 1021 patients with colon cancer, stages I-III, that were treated with a major resection with curative intent (R0 and R1). Of these patients, 487 (48%) were males and 534 were females, with mean ages of 71.7 (range: 32.9–91.2, SD: 10.6) and 72.8 (range: 20.3–99.6, SD: 11.1) years, respectively. The mean number of patients per calendar year increased from 17.5 patients/y in 1980–1989 to 38.7 patients/y in 2010–2016. The mean number of octogenarian patients per year increased from 3.6 to 9.3 patients, respectively. A laparotomy was performed in 974 (95.4%) patients compared to a laparoscopic procedure in 47 (4.6%) patients. Ten of the laparoscopic procedures were converted to open surgery.

**Table 4:** Characteristics of patients with colon cancer stages I-III that underwent major resections with curative intent

Characteristic	Total, *n* = 1021	Age group (years)						*P* value
		< 65, *n* = 233	65–74, *n* = 327	75–79, *n* = 201	80–84, *n* = 171	85–89, *n* = 66	≥ 90, *n* = 23	
Sex								0.043^a^
Females	534 (52)	121 (52)	156 (48)	105 (52)	99 (58)	38 (58)	15 (65)	
Males	487 (48)	112 (48)	171 (52)	96 (48)	72 (42)	28 (42)	8 (35)	
Calendar year								0.011^b^
1980–1989	175 (17)	50 (29)	61 (35)	28 (16)	27 (15)	9 (5)	0 (0)	
1990–1999	241 (24)	55 (23)	79 (33)	49 (20)	35 (15)	16 (7)	7 (3)	
2000–2009	334 (33)	82 (25)	92 (28)	64 (19)	67 (20)	18 (5)	11 (3)	
2010–2016	271 (27)	46 (17)	95 (35)	60 (22)	42 (16)	23 (9)	5 (2)	
ASA score								< 0.001^b^
1–2	591 (58)	190 (82)	215 (66)	94 (47)	69 (40)	19 (29)	4 (17)	
3	395 (39)	41 (18)	101 (31)	101 (50)	93 (54)	44 (67)	15 (65)	
4	35 (3)	2 (1)	11 (3)	6 (3)	9 (5)	3 (5)	4 (17)	
Localization								< 0.001^a^
Right colon	573 (56)	103 (44)	179 (55)	130 (65)	105 (61)	43 (65)	13 (57)	
Left colon	448 (44)	130 (56)	148 (45)	71 (35)	66 (39)	23 (35)	10 (43)	
Stage (TNM)								0.050^b^
I	154 (15)	33 (14)	54 (17)	25 (12)	27 (16)	12 (18)	3 (13)	
II	548 (54)	125 (54)	157 (48)	116 (58)	91 (53)	43 (65)	16 (70)	
III	319 (31)	75 (32)	116 (35)	60 (30)	53 (31)	11 (17)	4 (17)	
R-status								0.44^c^
R0—resection	983 (96.3)	223 (96)	317 (97)	196 (98)	160 (94)	64 (97)	23 (100)	
R0—resection with perforation	20 (2)	7 (3)	5 (2)	3 (2)	4 (2)	1 (2)	0 (0)	
R1—resection	18 (2)	2 (1)	4 (1)	2 (1)	7 (4)	1 (2)	0 (0)	
Type of resection								0.003^c^
Right hemicolectomy	504 (49)	87 (37)	156 (48)	113 (56)	99 (58)	37 (56)	12 (52)	
Transverse resection	24 (2)	5 (2)	9 (3)	4 (2)	2 (1)	4 (6)	0 (0)	
Left hemicolectomy	131 (13)	39 (17)	44 (14)	27 (13)	12 (7)	4 (6)	5 (22)	
Sigmoid and high anterior resections	267 (26)	75 (32)	89 (27)	41 (20)	43 (25)	16 (24)	3 (13)	
Hartmann's operation	35 (3)	10 (4)	12 (4)	5 (2)	3 (2)	3 (4)	3 (13)	
Subtotal resection	55 (5)	15 (6)	17 (5)	10 (5)	11 (6)	2 (3)	0 (0)	
Other resections	5 (1)	2 (1)	0 (0)	1 (1)	1 (1)	1 (2)	0 (0)	
Emergency surgery								0.13^a^
Yes	141 (14)	30 (13)	47 (14)	20 (10)	27 (16)	7 (11)	10 (43)	
No	880 (86)	203 (87)	280 (86)	181 (90)	144 (84)	59 (89)	13 (57)	

Values are the number of patients (%), unless otherwise indicated. ^a^Cochran-Armitage exact trend test; ^b^Ordinal logistic regression with age group as covariate; ^c^Nominal logistic regression with age group as covariate

### Postoperative complications and 90-day mortality after a major resection with curative intent

In 9.6% of cases, the Clavien-Dindo score was 3 or more. Anastomotic leakage was diagnosed in 2.5% (26/1021), and wound dehiscence in 1.7% (17/1021) of patients. A reoperation was required after 12.1% (17/141) of emergency resections, compared to 5.6% (49/880) of elective resections (*p* = 0.004). Table 5 presents the risk factors we identified that were associated with postoperative complications.

**Table 5:** Factors associated with postoperative complications^a^ after major resections with curative intent (R0 and R1); *n* = 1021^b^

Factors	Unadjusted odds ratio (95% CI)	*P* value	Adjusted odds ratio (95% CI)	*P* value
Age (years)				
< 65	1 (reference)		1 (reference)	
65—74	1.51 (1.08 to 2.11)	0.016	1.35 (0.94 to 1.96)	0.11
75 – 79	2.13 (1.47 to 3.08)	< 0.001	1.51 (0.99 to 2.28)	0.053
80—84	2.83 (1.93 to 4.17)	< 0.001	1.96 (1.27 to 3.03)	0.002
85 – 89	2.96 (1.73 to 5.03)	< 0.001	2.14 (1.18 to 3.87)	0.013
≥ 90	7.60 (3.11 to 18.58)	< 0.001	5.36 (2.11 to 13.61)	< 0.001
Female sex	0.99 (0.78 to 1.25)	0.95	1.15 (0.89 to 1.49)	0.28
Calendar year				
1980–1989	1 (reference)		1 (reference)	
1990–1999	0.46 (0.32 to 0.67)	< 0.001	0.44 (0.30 to 0.66)	< 0.001
2000–2009	0.43 (0.30 to 0.60)	< 0.001	0.50 (0.33 to 0.94)	0.001
2010–2016	0.47 (0.33 to 0.68)	< 0.001	0.61 (0.40 to 0.94)	0.025
ASA score				
1–2	1 (reference)		1 (reference)	
3	2.00 (1.56 to 2.56)	< 0.001	1.43 (1.08 to 1.90)	0.013
4–5	19.07 (9.56 to 38.05)	< 0.001	10.86 (5.19 to 22.73)	< 0.001
Emergency surgery	2.51 (1.75 to 3.60)	0.001	2.30 (1.57 to 3.39)	< 0.001
Anaemia (g/dL haemoglobin)				
Female ≥ 12.0, Male ≥ 13.0	1 (reference)		1 (reference)	
Female 10–11.9, Male 11–12.9	1.97 (1.47 to 2.64)	< 0.001	2.26 (1.64 to 3.11)	< 0.001
Female < 10, Male < 11	4.76 (3.53 to 6.43)	< 0.001	5.61 (4.01 to 7.84)	< 0.001
Surgery duration (minutes)				
< 90	1 (reference)		1 (reference)	
90–179	1.01 (0.73 to 1.38)	0.96	0.96 (0.67 to 1.39)	0.84
≥ 180	2.24 (1.48 to 3.38)	0.001	1.52 (0.89 to 2.58)	0.13
Blood loss (mL)				
0–200	1 (reference)		1 (reference)	
201–400	1.21 (0.88 to 1.65)	0.24	1.48 (1.04 to 3.11)	0.029
401–800	1.67 (1.20 to 2.32)	0.002	2.16 (1.45 to 3.21)	< 0.001
> 800	3.69 (2.41 to 5.65)	< 0.001	4.13 (2.44 to 7.01)	< 0.001

^a^Complications were classified according to Clavien-Dindo grades; ^b^Patients included those with stages I-III colon cancer during 1980–2016

Among patients with colon cancer stages I-III, mortality within 90 days after admission was 4.4% (45/1021). The 90-day mortality rates increased successively with increasing age. The rates were 0.4% among patients aged < 65 years, 9.3% in octogenarian patients, and 34.8% in patients above 90 years old (*p* < 0.001). Table 6 presents the factors we identified that were associated with death within 90 days.

**Table 6:** Factors associated with 90-day mortality after major resections with curative intent (R0 or R1); *n* = 1021^a^

Factor	Dead within 90 days (%)	Unadjusted odds ratio (95% CI)	*P* value	Adjusted odds ratio (95% CI)	*P* value
Age (years)					
< 65	1/233 (0.4)	┐			
65 – 74	8/327 (2.4)	├ 1 (reference)		1 (reference)	
75 – 79	6/201 (3.0)	┘			
80 – 84	13/171 (7.6)	4.09 (1.91 to 8.77)	< 0.001	3.90 (1.60 to 9.52)	0.003
85 – 89	9/66 (13.6)	7.85 (3.29 to 18.73)	< 0.001	10.72 (3.74 to 30.71)	< 0.001
≥ 90	8/23 (34.8)	26.52 (9.77 to 72.01)		19.76 (5.53 to 70.5)	< 0.001
Female sex		0.54 (0.29 to 0.9976)	0.049	0.51 (0.24 to 1.08)	0.077
Calendar year					
1980-1989	12/175 (6.8)	1 (reference)		1 (reference)	
1990-1999	12/241 (5.0)	0.71 (0.31 to 1.62)	0.42	0.49 (0.17 to 1.38)	0.18
2000-2009	10/334 (3.0)	0.42 (0.18 to 0.99)	0.048	0.29 (0.09 to 0.84)	0.022
2010-2016	11/271 (4.1)	0.57 (0.25 to 1.33	0.20	0.45 (0.17 to 1.20)	0.11
ASA score				3.77 (2.12 to 6.69)	< 0.001
1–2	10/591 (1.7%)	1 (reference)		1 (reference)	
3	20/395 (5.1%)	3.10 (1.43 to 6.69)	0.004	1.51 (0.63 to 3.59)	0.35
4	15/35 (42.9%)	43.58 (17.44 to 108)	< 0.001	12.60 (4.26 to 37.25)	< 0.001
Emergency surgery		10.24 (5.50 to 19.09)	< 0.001	6.80 (3.24 to 14.28)	< 0.001

^a^Patients included those with stages I-III colon cancer during 1980–2016; results are from a logistic regression analysis, with death as the dependent variable; unadjusted: performed with one covariate at a time; adjusted: performed with all listed covariates included simultaneously

### Long-term relative survival, local recurrence, and metastasis after a major resection with curative intent

**Fig. 2:**
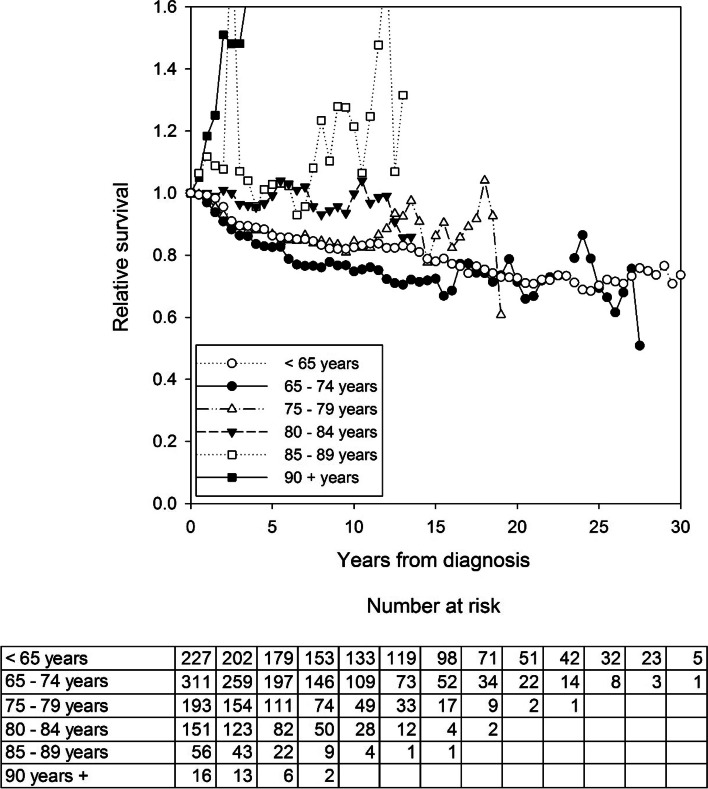
Relative survival after colon cancer resection with curative intent in patients that survived 90 days. Relative survival is stratified by age group. Table columns represent the number of patients at risk at surgery (time = 0) and every 2.5 years thereafter

Overall, the 5 year relative survival rate was 83.2% (95% CI: 79.4. to 86.8) for all patients with stages I-III disease that underwent major resections with a curative intent. Relative survival rates after a major resection with curative intent in patients who survived 90 days are presented in Fig. 2. Patients aged 65–74 years had the lowest 5 year relative survival rate: 79.0% (95% CI: 72.9 to 84.4), compared to 88.4% (95% CI: 77.0 to 99.1) in octogenarian patients.

When we excluded patients that died within the first 90 days, the overall 5 year relative survival rate was 87.5% (95% CI: 83.6 to 91.1). In this group, patients aged 65–74 years had the lowest 5 year relative survival rate: 81.2% (95% CI: 75.1 to 86.6), compared to 98.7% (95% CI: 86.5 to 110.0) in octogenarian patients.

Factors associated with relative long-term survival are presented in Table 7. Long-term relative survival rates did not differ significantly between the different age groups. A similar multivariable analysis performed in a selected group of patients with stage III colon cancer revealed that patients with left-sided colon cancer had better survival than those with right-sided colon cancer (OR = 0.55, 95% CI: 0.33 to 0.91; *p* = 0.02). This effect was not found in separate analyses of patients with stage I or stage II colon cancer.

**Table 7:** Factors associated with relative long-term relative survival, among patients that survived 90 days; *n* = 976^a^

Factor	Unadjusted hazard ratio (95% CI)	*P* value	Adjusted hazard ratio (95% CI)	*P* value
Age (years)				
< 65	1 (reference)		1 (reference)	
65—79	1.30 (0.84 to 2.03)	0.24	1.0005 (0.62 to 1.60)	0.998
≥ 80	0.58 (0.19 to 1.71)	0.32	0.73 (0.36 to 1.47)	0.38
Female sex	1.52 (0.98 to 2.36)	0.061	1.56 (1.03 to 2.36)	0.035
Calendar year				
1980–1989	1 (reference)		1 (reference)	
1990–1999	0.80 (0.47 to 1.35)	0.40	0.74 (0.44 to 1.24)	0.25
2000–2009	0.48 (0.28 to 0.85)	0.011	0.54 (0.31 to 0.93)	0.027
2010–2016	0.36 (0.18 to 0.70)	0.003	0.46 (0.25 to 0.85)	0.013
CCI	1.39 (1.14 to 1.68)	0.001	1.39 (1.12 to 1.73)	0.003
ASA score				
1–2	1 (reference)		1 (reference)	
3	1.85 (1.23 to 2.81)	0.003	1.64 (1.03 to 2.60)	0.036
4	5.45 (2.45 to 12.12)	< 0.001	4.79 (2.09 to 10.97)	< 0.001
Emergency surgery	3.07 (1.99 to 4.73)	< 0.001	2.13 (1.35 to 3.35)	0.001
Left vs. right colon	0.94 (0.62 to 1.43)	0.77	0.75 (0.49 to 1.14)	0.18
TNM-stage				
I	1 (reference)		1 (reference)	
II	3.92 (0.49 to 31.57)	0.20	1.98 (0.55 to 7.12)	0.30
III	16.81 (2.16 to 131)	0.007	8.17 (2.36 to 28.3)	0.001
R-status				
R0—resection	1 (reference)		1 (reference)	
R0—resection with perforation	9.78 (5.42 to 17.66)	< 0.001	4.81 (2.47 to 9.35)	< 0.001
R1—resection	3.54 (1.24 to 10.14)	0.018	3.25 (1.20 to 8.84)	0.021

^a^Patients included those with stages I-III colon cancer during 1980–2016, treated with a major resection with curative intent (R0 and R1). Results are from a multivariable analysis; unadjusted: performed with one covariate at a time; adjusted: performed with all listed covariates included simultaneously. *CCI* Charlson Comorbidity Index, classified as 0, 1, 2, or 3 +

Local recurrence was diagnosed in 4.4% (43/973) of patients. The overall estimated 5 year local recurrence rate was 4.5% (95% CI: 3.7 to 5.3). The estimated 5 year local recurrence rates after an R0 resection, an R1 resection, or a resection with tumour perforation were 4.3% (95% CI: 3.6 to 5.0), 43.2% (95% CI: 10.2 to 76.2), and 57.5% (95% CI: 19.1 to 95.9), respectively. The estimated 5 year local recurrence rates were not affected by age.

Metastatic disease was diagnosed in 20% (195/973) of patients. The overall estimated 5 year metastasis rate was 22.5% (95% CI: 19.5 to 25.5). The estimated 5 year local metastasis rates after an R0 resection, an R1 resection, or a resection with tumour perforation were 21.2% (95% CI: 18.2 to 24.2), 45.5% (95% CI: 17.9 to 73.1), and 70.4% (95% CI: 47.2 to 93.6), respectively. The estimated 5 year metastasis rates were not affected by age.

### Chemotherapy

Starting in 1993, adjuvant chemotherapy was given to 53% (72/137) of patients under 75 years of age that underwent a major resection with curative intent for stage III disease. Among these patients, 28% (16/58) received adjuvant chemotherapy in 1993–2004, and 71% (56/79) received adjuvant chemotherapy in 2005–2016. Among patients aged 75–84 years, a selected group of 13% (11/85) received adjuvant chemotherapy. Among patients treated with a major resection with curative intent for stage II disease, 7% (15/214) received adjuvant chemotherapy.

Among patients that underwent palliative surgery or best supportive care, 34.5% (158/458) received palliative chemotherapy. This rate remained stable throughout the study period. The percentage of patients given palliative chemotherapy decreased as age increased. Palliative chemotherapy was given to 76% (79/104) of patients < 65 years, compared to 2.7% (4/148) of octogenarian patients.

## Discussion

In this series, the rate of patients selected for surgical treatment decreased as patient age increased. Nevertheless, postoperative morbidity and 90-day mortality rates increased as patient age increased. During the study period, the percentage of octogenarian patients that underwent a major resection with curative intent increased, and the 90-day mortality was reduced. However, among patients that survived the first 90 days, long-term relative survival was independent of age.

### All patients

Previous studies have pointed out age-related disparities in multimodal cancer treatments [9, 20]. In patients with colon cancer, individual treatment plans are based on accurate disease staging. In the first period (1980–1989) of the present study, we observed a transient trend towards a higher proportion of older patients with unknown disease stages. During the study period, we found significant progress in staging availability and precision, and focus was placed on the importance of preoperative staging, irrespective of patient age. Nevertheless, the proportion of patients with unknown stages among octogenarian patients in this series was low, compared to the proportions based on national data from several European countries [21]. Moreover, the disease stages at admission were equally distributed across the age groups, and the proportion of patients that presented with stage IV disease (25%) was comparable to proportions reported previously [22, 23].

Surgery is the cornerstone of colon cancer treatment. The primary objective of surgery is either radical resection or endoscopic resection, for early-stage tumours. Palliative surgery may be indicated as part of a multimodal treatment in patients with advanced disease or in cases with obstruction. Overall, the percentage of patients that underwent surgical treatments in this series was 89%. This percentage decreased as age increased. Surgery was performed in 93% of patients younger than 80 years and 82% of octogenarian patients. These findings were comparable to national data from European countries, where surgical treatment rates ranged between 59 and 79% among patients 80 years and older [21]. Variations in the overall rates of patients that undergo surgical treatment for colon cancer among different series are likely to depend on demographic, socioeconomic, and clinically related factors. The availability of healthcare services in our catchment area was high, and the threshold for referring patients to the hospital, irrespective of age, was low. However, because comorbidity increased with age, the rate of patients considered unsuitable for surgical treatment was relatively high among older patients.

The overall rate of patients that underwent emergency surgery in this series was 16%, and the rate increased with increasing age. Previous studies have shown significant variability (8–34%) in the rates of emergency surgery; these differences might be due to differences in the definition of emergency surgery and the selection of patient cohorts [24–26]. The rate of emergency surgery in this series was lower than the 25% reported previously, in a comparable population-based study from Sweden [27]. We observed that the rate of emergency surgery declined throughout the 37 years of the study. This finding might be related to a continuous increase in the availability of health care services, including the implementation of fast-track examinations, when alarm symptoms indicated colorectal cancer, and a higher societal awareness of this disease.

In parallel with the increases in population aging and the number of older patients admitted to hospital with colon cancer, the rate of octogenarian patients that underwent surgery increased. Hence, the proportion of octogenarian patients considered eligible for surgery has increased. A comparison of general health between the current and previous generations is difficult to assess objectively, and we lack evidence that older people in the current generation are healthier than those in previous generations [28, 29]. However, comorbid disease treatments and perioperative care have improved during the last few decades, and these advances have lowered the threshold for surgery [30–32].

The literature has shown variability in the rates of short-term mortality among patients with colon cancer. Clearly, differences in patient populations and differences in patient selection procedures for different treatment options, primarily surgical treatments, have major impacts on the outcome. In the present study, the overall 90-day mortality was 13.5%, and it increased, with increasing age, to 22.4% among octogenarian patients. These rates were comparable to rates reported in other unselected population-based series [26, 33]. We found that comorbidity, advanced TNM-stages, and emergency surgery had profound negative effects on the 90-day mortality. These associations were consistent with those demonstrated in previous reports [34, 35]. We noted a 48% reduction in the overall 90-day mortality rate, between the first and last decades of the observational period. The basis for this improvement was multifactorial, but it was driven by the general, continuous progress in medical treatments during the study period. Although we observed a significant increase in short-term mortality with increasing age, the long-term relative survival rates of young and old patient groups converged over time, and after 5 years, survival was independent of age. The 5 year relative survival among all patients was 58.5%, comparable to rates reported in previous studies on unselected series of patients with colon cancer [36].

### Patients with stages I-III disease that underwent a major resection with curative intent

Among patients with stages I-III disease at diagnosis, 92.6% (1021/1102) were treated with a major resection with curative intent, comparable to the proportions reported previously in studies on colon cancer [37]. Although the rate was lower among octogenarian patients (90.1%, 237/263), it was similar to the overall rate, which indicated that the approach to surgical treatment remained consistent, irrespective of age. During the first part of this study, the selection of patients for a major resection with curative intent was performed by a traditional interdisciplinary team, which included the surgeon and the anaesthesiologist. This selection was primarily based on a clinical evaluation combined with the ASA-score. Later, the focus changed, and treatment decisions were increasingly performed by multidisciplinary teams, which also included oncologists, radiologists, and pathologists [5].

The overall rate of postoperative morbidity, defined as a Clavien-Dindo score of 3 or more, was 9.6%, and the overall 90-day mortality was 4.4%. We observed a significant reduction in both postoperative morbidity and mortality during the study, and as in other series, we confirmed that high ASA scores and the need for emergency surgery had negative impacts on both endpoints. Moreover, high peri-operative blood loss increased the postoperative morbidity, which highlighted the importance of the surgical technique [38]. Finally, preoperative anaemia was significantly associated with an increased risk of postoperative complications. In a previous meta-analysis by Fowler et al., preoperative anaemia was also associated with a poor postoperative outcome [39]. Accordingly, methods for detecting and treating preoperative anaemia would be beneficial.

The major challenge in treating colon cancer, which was noted in this series and confirmed by others, is the significant increase in postoperative morbidity and mortality with increasing age, even after a thorough patient selection process. In this series, octogenarian patients selected to undergo major curative surgery had a significantly increased risk of postoperative morbidity and mortality compared to younger patients. The mortality rate was 0.4% among patients aged < 65 years, and it increased by 25-fold, to 10.1%, in octogenarian patients.

Nevertheless, the 5 year relative survival rate in this series was equivalent across age groups, consistent with findings in previous series [36, 40–42]. Among patients that survived 90 days after surgery, long-term survival was most significantly negatively impacted by the TNM stage, the R-status, and the presence of a tumour perforation [36, 40–42]. As observed previously [36, 40–42], the negative effect of emergency surgery persisted past the postoperative period. This finding highlighted the need to enhance the focus and follow-up for this group of patients.

As the population ages, octogenarian patients will become the most common group with colon cancer. Consequently, measures are needed to reduce the excess rates of postoperative morbidity and mortality among older patients. Increasing the focus on the process of selecting patients to different levels of treatment will be highly important, both for the individual patient and for the healthcare system. It is essential to perform geriatric assessments systematically in the preoperative work-up [43–45], pay attention to the concept of prehabilitation [46], and increase focus on patient preferences [47]. Recent reports have demonstrated the value of a geriatric assessment in summarizing the patient's degree of frailty and predicting postoperative morbidity and mortality for older patients with colon cancer [48]. The Society for Geriatric Oncology has recommended these assessments for all patients with cancer that are over 70 years of age [49]. In a systematic review, more than half of older patients with cancer were considered to be in a pre-frailty or frailty condition [50], and both these conditions were associated with adverse postoperative outcomes.

Most efforts to reduce postoperative morbidity and mortality rates have focused on the peri-operative and immediate postoperative statuses. Thus, the concept of prehabilitation prior to surgery has not gained sufficient attention. As part of this concept, the geriatric assessment evaluates several individual modifiable factors relevant to status optimization prior to surgery [51]. Moreover, a multidisciplinary team approach was shown to improve the postoperative outcome in frail patients [52]. Currently, an ongoing prospective multicentre study is examining multimodal prehabilitation for patients with colorectal cancer. Hopefully, those results will provide valuable information regarding the role of prehabilitation in the future management of older patients with cancer [46].

Numerous factors contribute to heterogeneity in the group of older patients with cancer. It is important to consider that personal patient preferences regarding treatment decisions might vary substantially among older patients. In the late stages of life, some needs, like preserving the remaining quality of life, may outweigh the need for radical treatment [47, 53]. It has been shown that the physician’s recommendation was the most decisive factor in influencing the patient’s decision [54]. That finding emphasized the importance of a thorough, and preferably evidence-based, foundation for the physician’s advice.

### Strengths and weaknesses

The main strength of this study was the transparent presentation of a consecutive, population-based cohort of patients with colon cancer that were treated in accordance with current evidence-based guidelines over a period of 37 years. Our institution was the primary hospital for a stable population throughout this extensive observational period, and thus, the cohort was suitable for evaluating trends over time. We believe that octogenarian patients with colon cancer will emerge as an important entity; thus, the results from this series provide important contributions to the current state of the field.

The main limitation of the study was its retrospective design. Due to its observational nature, we could not investigate causality. Moreover, the results may not be applicable to the older population, in general. Frail and unfit patients might not have been referred to our hospital, due to their clinical status. Finally, unknown or unrecorded confounders might have affected decisions regarding patient selection and treatment.

## Conclusion

This study showed that octogenarian patients treated for colon cancer had adverse 90-day mortality rates, but among those that survived 90 days postoperatively, the long-term survival rate was equivalent to that of younger patients. The increasing fraction of older patients in years to come will become a major challenge in treating colon cancer. In addressing that challenge, early disease detection, followed by prehabilitation, a multidisciplinary approach with a geriatric assessment, and a meticulous post-operative follow up will be essential factors for improving treatment results and surmounting current standards.

## Data Availability

The dataset used for this study is located on a secure server in the Levanger Hospital data system. The database was confirmed by comparing data with corresponding data in the Norwegian Cancer Registry 1980–2016 (https://www.kreftregisteret.no). The data are not publicly available as their containing information could compromise the privacy of research participants.

## Abbreviations

ASA: American Society of Anaesthesiology
CCI: Charlson Comorbidity Index
CI: Confidence interval
SD: Standard deviation
